# Regulatory and Enterotoxin Gene Expression and Enterotoxins Production in *Staphylococcus aureus* FRI913 Cultures Exposed to a Rotating Magnetic Field and *trans*-Anethole

**DOI:** 10.3390/ijms23116327

**Published:** 2022-06-06

**Authors:** Paweł Kwiatkowski, Aleksandra Tabiś, Karol Fijałkowski, Helena Masiuk, Łukasz Łopusiewicz, Agata Pruss, Monika Sienkiewicz, Marcin Wardach, Mateusz Kurzawski, Sebastian Guenther, Jacek Bania, Barbara Dołęgowska, Iwona Wojciechowska-Koszko

**Affiliations:** 1 Department of Diagnostic Immunology, Pomeranian Medical University in Szczecin, Powstancow Wielkopolskich 72, 70-111 Szczecin, Poland; iwona.wojciechowska.koszko@pum.edu.pl; 2 Department of Food Hygiene and Consumer Health Protection, Wroclaw University of Environmental and Life Sciences, C.K. Norwida 31, 50-375 Wroclaw, Poland; aleksandra.tabis@upwr.edu.pl (A.T.); jacek.bania@upwr.edu.pl (J.B.); 3 Department of Microbiology and Biotechnology, Faculty of Biotechnology and Animal Husbandry, West Pomeranian University of Technology, Piastow 45, 70-311 Szczecin, Poland; 4 Department of Medical Microbiology, Pomeranian Medical University in Szczecin, Powstancow Wielkopolskich 72, 70-111 Szczecin, Poland; h.masiuk@op.pl; 5 Center of Bioimmobilisation and Innovative Packaging Materials, Faculty of Food Sciences and Fisheries, West Pomeranian University of Technology, Janickiego 35, 71-270 Szczecin, Poland; lukasz.lopusiewicz@zut.edu.pl; 6 Department of Laboratory Medicine, Pomeranian Medical University in Szczecin, Powstancow Wielkopolskich 72, 70-111 Szczecin, Poland; agata.pruss@pum.edu.pl (A.P.); barbara.dolegowska@pum.edu.pl (B.D.); 7 Department of Pharmaceutical Microbiology and Microbiological Diagnostic, Medical University of Lodz, Muszynskiego 1, 90-151 Lodz, Poland; monika.sienkiewicz@umed.lodz.pl; 8 Faculty of Electrical Engineering, West Pomeranian University of Technology, Sikorskiego 37, 70-313 Szczecin, Poland; marcin.wardach@zut.edu.pl; 9 Department of Experimental and Clinical Pharmacology, Pomeranian Medical University in Szczecin, Powstancow Wielkopolskich 72, 70-111 Szczecin, Poland; mateusz.kurzawski@pum.edu.pl; 10 Pharmaceutical Biology, Institute of Pharmacy, University of Greifswald, Friedrich-Ludwig-Jahn-Straße 17, 17489 Greifswald, Germany; sebastian.guenther@uni-greifswald.de

**Keywords:** rotating magnetic field, *trans*-anethole, regulatory gene, staphylococcal enterotoxin gene, staphylococcal enterotoxins, *Staphylococcus aureus*

## Abstract

The study aimed to examine the influence of a rotating magnetic field (RMF) of two different frequencies (5 and 50 Hz) on the expression of regulatory (*agrA*, *hld*, *rot*) and staphylococcal enterotoxin (SE—*sea*, *sec*, *sel*) genes as well as the production of SEs (SEA, SEC, SEL) by the *Staphylococcus aureus* FRI913 strain cultured on a medium supplemented with a subinhibitory concentration of *trans*-anethole (TA). Furthermore, a theoretical model of interactions between the bacterial medium and bacterial cells exposed to RMF was proposed. Gene expression and SEs production were measured using quantitative real-time PCR and ELISA techniques, respectively. Based on the obtained results, it was found that there were no significant differences in the expression of regulatory and SE genes in bacteria simultaneously cultured on a medium supplemented with TA and exposed to RMF at the same time in comparison to the control (unexposed to TA and RMF). In contrast, when the bacteria were cultured on a medium supplemented with TA but were not exposed to RMF or when they were exposed to RMF of 50 Hz (but not to TA), a significant increase in *agrA* and *sea* transcripts as compared to the unexposed control was found. Moreover, the decreased level of *sec* transcripts in bacteria cultured without TA but exposed to RMF of 50 Hz was also revealed. In turn, a significant increase in SEA and decrease in SEC and SEL production was observed in bacteria cultured on a medium supplemented with TA and simultaneously exposed to RMFs. It can be concluded, that depending on SE and regulatory genes expression as well as production of SEs, the effect exerted by the RMF and TA may be positive (i.e., manifests as the increase in SEs and/or regulatory gene expression of SEs production) or negative (i.e., manifests as the reduction in both aforementioned features) or none.

## 1. Introduction

*Staphylococcus aureus* is an opportunistic microorganism able to induce infection in both out- and inpatients [1]. These infections mainly affect the skin, soft tissues, and bones [2]. The virulence of *S. aureus* can be manifested by several factors, both intracellular and secreted to the external environment [3]. These are primarily surface proteins of antigenic nature, responsible for adherence, as well as many enzymes and toxins that enhance the infection process [4]. Staphylococcal enterotoxins (SEs) belong to a large heterogeneous group of exotoxins and include 23 serologically different proteins (marked with letters from SEA to SElY) [5]. SEs are responsible for staphylococcal food poisoning, show significant thermostability, and are resistant to many proteolytic enzymes, such as pepsin and trypsin, thanks to which they remain active in the digestive tract [6]. The expression of virulence factors and primary metabolism genes are affected by a variety of mechanisms [7,8]. The regulatory system can directly affect pathogen-associated genes and can be simultaneously controlled by other mechanisms [9]. The relationships between individual regulatory systems are still unexplained, but they certainly determine the ability to adapt microorganisms to various environmental conditions and also have a decisive impact on the intensity of the infection process. The functioning of regulatory systems depends on many factors, such as pH, atmosphere, cell growth phase, and density of bacterial populations [10].

Received signals from the external environment are primarily responsible for two-component regulatory systems, consisting of a sensor and regulatory protein [11,12]. One of them is the Agr system encoded by chromosomal genes, whose structure is based on a DNA fragment (3 kb) encoding two transcripts (RNAII and RNAIII). The RNAII transcript is encoded by four genes (*agrA*, *agrB*, *agrC,* and *agrD*) whose functions are substantially different [13]. AgrA is a regulatory protein and transcription factor, the agrC protein is an autophosphorylating histidine kinase anchored in the cell membrane, agrB is a transport protein also involved in the post-translational modification of the agrD protein, and the agrD protein is an extracellular secreted autoinducing peptide (AIP), which is also a specific ligand for the protein of the quorum-sensing system [14]. The RNAIII transcript is encoded by the *hld* gene, which regulates, i.e., the activation and repression of the expression of *agr* system genes and several genes responsible for virulence [15]. The expression of genes encoding virulence of *S. aureus* also depends on the bacterial growth phase [16]. Logarithmic growth is associated with the production of proteins that condition successful colonization, forming part of cell surface structures (protein A, collagen-binding protein, fibronectin-binding protein, fibrinogen binding protein) and adhesions [17]. Expression of genes encoding *S. aureus* virulence factors can also be regulated by other systems, such as mechanisms of positive and negative gene regulation involving antisense RNA, transcription factors (RNAIII), SarA family proteins, as well as MgrA, Rot, SigmaB, which they bind to DNA at gene promoter sites, activating or inhibiting their transcription [18].

*trans*-Anethole (TA) is a major compound of many essential oils (EOs), including fennel (*Foeniculum vulgare* Mill.), anise (*Pimpinella anisum* L.), or star anise (*Illicium anisatum* L.) [19,20]. TA (due to its wide availability) is used in many industries, such as perfume, pharmaceutical, as well as food [21]. Furthermore, it possesses many interesting properties, including antimicrobial, which were confirmed in our previous studies as well as by other authors [21,22,23]. TA was also proved to demonstrate a lack of toxicity at low doses, and it was reaffirmed as Generally Recognized as Safe (GRAS) [24].

Magnetic fields (MFs) can be divided into two main types: direct-current magnetic field (DCMF) and alternating current magnetic field (ACMF). DCMF does not vary in time, or it changes very slowly. This type of MF does not have a frequency (the MF vector is constant in time and space). Static MF (SMF) is an example of DCMF [25,26,27,28]. In contrast to DCMF, ACMF varies in frequency. Pulsating MF that changes as a sine-wave with time at each point in space is an example of ACMF [29,30,31,32,33]. A superposition of two or more ACMFs of identical frequency but spatially displaced in phase with respect to one another causes rotation of the MF (RMF). RMF changes over time and can be characterized by its frequency [34].

The idea of gene expression, investigated in *S. aureus* under RMF and TA influence, originated from previous research performed by Fijałkowski et al. [35] and Qiu et al. [36]. The studies showed that RMF [35] and fennel essential oil (FEO), which contains a high level of TA [36], decreased the expression of genes encoding selected SEs. However, the studies on the influence of simultaneous exposure of *S. aureus* to RMF and TA at the same time on changes in SEs genes expression have not been published so far. Hence, the study aimed to investigate the influence of the RMF of two different frequencies (5 and 50 Hz) on the regulatory (*agrA*, *hld*, *rot*), as well as SE (*sea*, *sec*, *sel*) gene expression in *S. aureus* FRI913, cultured on a medium supplemented with a subinhibitory concentration of TA. Additional attention was paid to a possible effect of RMF and/or TA on the production of SEs (SEA, SEC, SEL). Moreover, a theoretical model explaining possible interactions between the RMF-activated bacterial medium and bacterial cells was proposed.

## 2. Results

### 2.1. Evaluation of Antibacterial Activity of TA

Based on the agar dilution method, it was observed that TA showed antibacterial activity against the *S. aureus* FRI913 strain. The obtained minimum inhibitory concentration (MIC_100_) and subinhibitory concentration (MIC_50_) values of TA were 49.4 ± 0.0 mg/mL and 24.7 ± 0.0 mg/mL, respectively. It was also confirmed that an addition of 1% (*v*/*v*) Tween 80 in the MHA medium did not influence the viability of *S. aureus* FRI913 strain.

### 2.2. Selection of Housekeeping Gene

Among the tested housekeeping genes, the r-value of *16S rRNA* (0.930) was the closest to 1. Thus, this gene appeared to be the most stable gene among all the analyzed genes (*16S rRNA, rpoB*, *gyrB*, *recA*, *rho*, *pta*, *rplD*, and *tpo*). Detailed statistical data of all housekeeping genes are listed in Supplementary Materials, Table S1.

### 2.3. Effect of RMF and TA on SEs and Regulatory Gene Expression

The *S. aureus* reference strain was screened for changes in the expression of *sea, sec, sel* genes exposed to RMF and/or TA. Detailed data on the quantification of the analyzed genes are listed in Supplementary Materials—Table S2.

**Figure 1:**
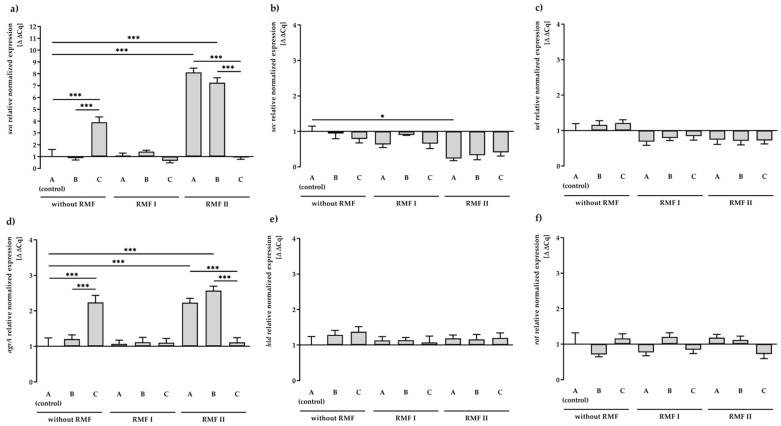
Relative expression levels of SEs: *sea* (**a**), *sec* (**b**), and *sel* (**c**) and regulatory: *agrA* (**d**), *hld* (**e**), *rot* (**f**) genes in *S. aureus* FRI913 grown on Mueller–Hinton agar non-supplemented with *trans*-anethole (A), supplemented with 1% (*v*/*v*) Tween 80 (B), and supplemented with 1% (*v*/*v*) Tween 80 and subinhibitory concentration of *trans*-anethole (C), exposed to rotating magnetic fields (RMFs: RMF I: 5 Hz, RMF II: 50 Hz) and unexposed (without RMF). Control—bacteria cultured on a medium non-supplemented with *trans*-anethole (A) and unexposed to RMF. Data are shown as the mean ± standard deviation. * *p* < 0.05; *** *p* < 0.001.

As shown in Figure 1a, transcription of the *sea* gene did not differ statistically significantly between RMF of 5 Hz and unexposed control settings. In contrast, a significant increase (*p* < 0.001) in the *sea* gene expression (approx. 8-folds) was found due to exposure to the RMF of 50 Hz. Similarly, a significant increase (*p* < 0.001) in gene expression of *sea*, compared to the control (medium A unexposed to RMFs), was observed when bacteria were incubated on a medium with TA (approx. 4-folds). Nevertheless, the presence of RMF of 50 Hz and TA contributed to a significant decrease (*p* < 0.001) in the transcript level of *sea*. It was also found that the exposure to RMF (both frequencies) decreased the expression level of *sec* and *sel* genes (Figure 1b,c). However, in this case, only exposure to the RMF of 50 Hz resulted in significant changes (*p* < 0.05) in gene expression of *sec* in comparison to the control.

The influence of the RMF and/or TA on the expression of regulatory genes (*agrA*, *hld*, *rot*) (Figure 1d–f) was also investigated. Detailed data on the quantification of the analyzed genes are listed in Supplementary Materials, Table S2. In our study, after 24 h of incubation, we observed a significant increase (*p* < 0.001) in the transcript level of the *agrA* gene in culture on TA-supplemented medium or culture exposed to the RMF of 50 Hz. Similar to the expression of the *sea* gene, the presence of RMF of 50 Hz and TA contributed to a significant decrease (*p* < 0.001) in the transcript level of *agrA*. The *agrA* gene was the only regulatory gene among those analyzed that showed significant differences in expression compared to the control (medium A unexposed to the RMFs). However, the level of upregulation was low and did not correlate with the expression of the dependent *sec* gene.

It was also observed that 1% Tween 80 (medium B) had no effect on the expression of all tested SE genes, excluding *agrA* and *sea* genes’ exposure to RMF of 50 Hz.

### 2.4. Effect of RMF and TA on SEs Production

**Figure 2:**
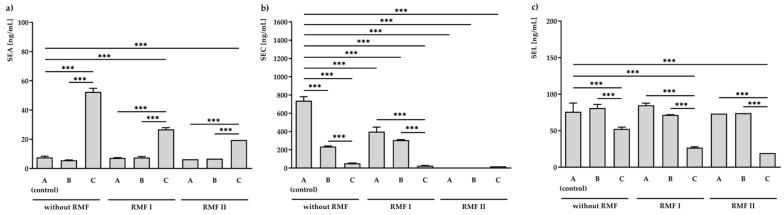
ELISA analysis of staphylococcal enterotoxins: SEA (**a**), SEC (**b**), and SEL (**c**) production in *S. aureus* FRI913 grown on Mueller–Hinton agar non-supplemented with *trans*-anethole (A), supplemented with 1% (*v*/*v*) Tween 80 (B), and supplemented with 1% (*v*/*v*) Tween 80 and subinhibitory concentration of *trans*-anethole (C), exposed to rotating magnetic fields (RMFs: RMF I: 5 Hz, RMF II: 50 Hz) and unexposed (without RMF). Control—bacteria cultured on a medium non-supplemented with *trans*-anethole (A) and unexposed to RMFs. Data are shown as the mean ± standard deviation. *** *p* < 0.001.

Bacteria grown on medium A and exposed to the RMF (5 and 50 Hz) was found to produce a similar amount of SEA (6.3 ± 0.7–7.6 ± 0.8 ng/mL). In accordance with the level of the transcript (Figure 2a), a significantly higher (*p* < 0.001) concentration of SEA (52.4 ± 2.5 ng/mL) was found in the *S. aureus* incubated on medium C (but not exposed to RMFs). Further exposure to RMF led to a decrease in SEA production to 26.8 ± 1.2 ng/mL for 5 Hz RMF treatment and 19.5 ± 0.6 ng/mL for 50 Hz RMF treatment (Figure 2a).

A significant effect of the investigated conditions was also observed in SEC concentrations (Figure 2b). The highest inhibition effect was observed in *S. aureus* incubated on medium A and exposed to the RMF of 50 Hz. In this condition, no production of SEC was noticed. Bacteria incubated on medium C and unexposed to RMFs produced 52.4 ± 2.5 ng/mL of SEC. This concentration was approximately fourteen times lower than that of the control (medium A unexposed to RMFs; 737.8 ± 43.2 ng/mL). Moreover, the presence of both RMF and TA contributed to even a higher reduction in the SEC production: 26.8 ± 1.2 ng/mL and 19.5 ± 0.6 ng/mL, respectively, for RMFs of 5 and 50 Hz.

Moreover, when the bacteria were cultured on a medium supplemented with TA but were not exposed to RMF (52.4 ± 2.5 ng/mL) or when they were exposed to RMF of 5 Hz (26.8 ± 1.2 ng/mL) or 50 Hz (19.5 ± 0.6 ng/mL) and TA, a significant decrease (*p* < 0.001) in SEL production as compared to the unexposed control (76.0 ± 11.8 ng/mL) was found.

It was also observed that 1% Tween 80 (medium B) did not affect the production of all tested SEs, excluding SEC. There was a statistically significant reduction (*p* < 0.001) in SEC production compared to the unexposed control.

In the used model, after 24 h of incubation, the production of SEA was mRNA dependent and regulated at the level of transcription in culture unexposed to the RMF of 5 Hz. Similar relationships between protein production and mRNA expression level were observed in the case of SEC without the use of RMFs. Expression at the level of the SEL protein was not regulated after this time at the level of transcription (Supplementary Materials, Table S3).

## 3. Discussion

The results of basic microbiological studies presented in this article were an extension of the previous studies in this area presented in the cited reference works [34,35,37,38,39,40]. The cited experimental studies were focused on the influence of the interaction between RMF and antibiotics, as well as the analysis of bacterial colony growth dynamics, the metabolic activity of cells, as well as genetic activity of selected bacterial species exposed to RMF. Nevertheless, a combined effect of TA and RMF on regulatory and SEs gene expression as well as on the concentration of selected SEs in *S. aureus* FRI913 strain has not been published so far. Moreover, our study is also the first to show the effect of TA on the above-mentioned analyzed parameters.

**Figure 3:**
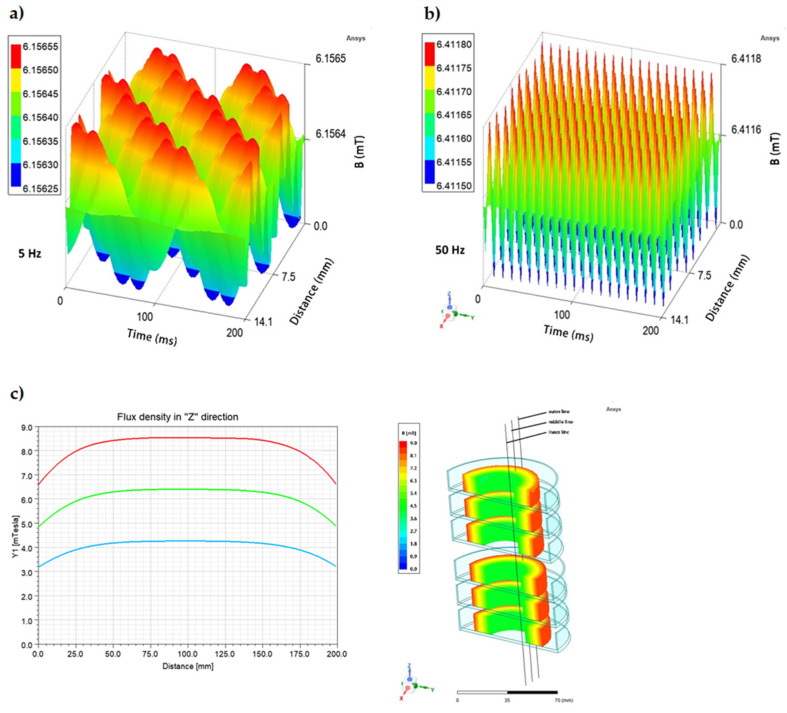
Changes in the magnetic flux characteristics depending on the applied AC frequency: (**a**) 5 Hz; (**b**) 50 Hz, and (**c**) spatial distribution of magnetic field in a cross-section (h = 20 cm) of RMF-bioreactor in the outer, middle, and inner zone from which bacteria were harvested for individual analyses.

It is widely recognized that the effect of the magnetic field is to a major extent related to its frequency and intensity [34,37,41,42,43,44] because these two factors determine the physical characteristics of the magnetic signal [45,46]. Therefore, a possible influence of these variables was also analyzed with regard to observed changes in the expression of regulatory genes (*agrA*, *hld,* *rot*) and genes encoding staphylococcal enterotoxins (SEs: *sea*, *sec*, *sel*), as well as the concentration of produced SEs. In the case of the RMF set-up used in the current study, the frequency of AC determines MF intensity as well as the physical characteristics of the magnetic wave shape. Therefore, the AC frequencies of 5 Hz (the lowest current frequency that can be used in the setup) and 50 Hz (the highest current frequency that can be used in the setup) at which the RMF was generated allowed obtaining MF of different parameters. At 5 Hz, the amplitude of the RMF was characterized by a longer period between maximal/minimal magnetic induction (*B*) states (100 ms) with *B*_average_ 6.16 mT as compared to the RMF generated at 50 Hz (10 ms) with *B*_average_ 6.41 mT (Figure 3a,b). It should also be noted, that the RMF was axially symmetric (Figure 3c) and magnetic field lines rotated in a horizontal direction, with the rotation frequency equal to the frequency of the electric current, as can be seen in the simulations: 5 Hz: https://www.youtube.com/watch?v=sTpGK0bRJt8 (accessed on 1 January 2020) and 50 Hz: https://www.youtube.com/watch?v=_uLUocd1c9k (accessed on 1 January 2020).

In our study, the influence of active factors such as RMF and TA on the expression level of regulatory (*agrA*, *hld*, *rot*) and SE (*sea*, *sec, sel*) genes’ expression as well as SE (SEA SEC, SEL) production was investigated. The expression of genes encoding *S. aureus* virulence factors can be regulated by several systems [47]. The primary metabolism gene expression depends on many factors, such as pH, atmosphere, cell growth phase, and density of bacterial populations [10].

In our experimental study, RMF and TA were shown to be important additional factors controlling bacterial metabolism. Significant differences were observed depending on three main quantities, i.e., the induction value of RMF, TA content, and RMF exposure time of a sample.

Relationships between individual regulatory systems are still unexplained but they certainly determine the ability of microorganisms to adapt to various environmental conditions and also have a decisive impact on the intensity of the infection process [10].

The results presented in the current study suggest that the level of gene expression and SEs production depends on different frequencies of the RMF associated with TA. The relative normalized numerical statistical data indicated a significant increase in *agrA* (*p* < 0.001) gene expression observed due to exposure to the RMF of 50 Hz in comparison to the control (medium non-supplemented with TA and unexposed to RMFs). In addition, obtained results showed that the frequency of RMF did not affect *rot* and *hld* genes. Moreover, a significant increase and decrease, respectively, in *sea* (*p* < 0.001) and *sec* (*p* < 0.05) gene expression under this condition were observed. This fact revealed that only by the application of the RMF of 50 Hz can *sea* gene expression and production of SEA be controlled.

According to available data, there are several reports presenting the effects of MFs on gene expression in bacteria. Potenza et al. [48] proved that SMF (300 mT) induced cell proliferation and changes in *Escherichia coli* gene expression. Moreover, Giorgi et al. [49] observed that mini-Tn10 and Tn5 transposition decreased under sinusoidal MF and increased under pulsed-square MF. Finally, Fijałkowski et al. [35] reported a lower expression level of *sea*, *seb*, *sec*, *sed*, and *see* genes under RMF influence compared to non-exposure samples. Interestingly, these authors observed lower expressions of the *sea* gene compared to our data. These results may coincide due to the incubation conditions. In the current study, we used *S. aureus* cultured on MHA, whereas, in the mentioned study, broth culture was performed. According to our observations, due to the occurrence of an ouzo effect of TA in combination with MHB and difficulty in reading MIC by the microdilution method, we decided to immobilize TA in a solid medium (MHA). Nevertheless, the observed *sec* gene expression level was similar in both studies.

In the current study, an increase in the transcript level of the *agrA* gene in *S. aureus* exposed to TA was observed. It is the only regulatory gene among the ones tested which showed significantly different expression levels in comparison to the control (medium non-supplemented with TA and unexposed to RMFs). The level of upregulation observed was high and could correlate with the expression of the *sea* gene as well as SEA production. What is more, there was no effect of TA on the *sec* expression gene. However, SEC production was lower (*p* < 0.001) than in the control. As stated in the literature, the expression of selected SEs could be influenced by subinhibitory concentrations of many natural compounds. For instance, Azizkhani et al. [50] revealed lower relative expression of *sea*, *sec*, *see,* and *agrA* in *S. aureus* ATCC 29213 cultured with a subinhibitory concentration of *Zataria mulitflora* Boiss. EO after 72 h in comparison to the control (without EO). Interestingly, data were obtained by Qiu et al. [36]. According to these studies, FEO (containing 88.91% of TA) in MHB reduced relative expression levels of *hla*, *sea*, *seb*, *tst,* and *agrA*, as well as the production of SEA, SEB, toxic shock syndrome toxin (TSST), and α-hemolysin. Results of the current study allow concluding that in addition to culture conditions, the presence of other compounds in FEO can also affect the expression/production level of the above-mentioned virulence factors. The influence of the EO solvent (dimethyl sulfoxide) used by these authors cannot be excluded here, either. Moreover, similar results were observed in other studies where the production of selected exotoxins, such as α-toxin, SEA, SEB, and TSST, in *S. aureus* was decreased by perilla [51], peppermint [52] EOs, or even thymol [53].

The above-mentioned results could be associated with the *S. aureus* Agr system involved in the regulation of SEs expression, which response to staphylococcal cell population density is known as quorum sensing [54]. The *sea* and *sec* genes products are well characterized and are well known for their superantigenic activity and ability to evoke emesis [55]. However, little is known about the *sel* gene, which is quite often clustered with the *sec* gene on a pathogenicity island SaPI in *S. aureus* [56,57]. However, the obtained results demonstrated a significant decrease in the *sea* gene expression and SEC production in the reference strain under TA and RMF (50 Hz) influence, but further research is needed to understand this mechanism.

It can be assumed that the impact of RMF on a microbial sample corresponds to the impact of this field on the free surface of the medium and on bacterial cells. It should be emphasized, that previously published studies concerning changes in the metabolism of microorganisms exposed to RMF in the context of the final effect and not the state of the medium with organic substance (OS) on the free surface [34,35,37,38]. Hence, this observation implies that either bacteria were sensitive to RMF or OS in the medium had magnetic properties, and the interaction between MF and the medium affected microorganisms on the surface of the medium.

In the theoretical model, we assumed, that both OS on the free surface of the medium and bacteria cultured on the medium were sensitive to RMF as well as to other possible external physical factors. For this purpose, we identified a microbial sample as a three-phase system containing bacterial colonies and a multicomponent bacterial medium which were combined and simultaneously separated by a third phase called the pseudo surface phase (PSP). Additionally, the medium was physically defined as a solid and flexible substrate containing a mass of OS in the form of small irregularly shaped particles (POS). During the preparation of the bacterial medium, the OS particles were randomly dispersed throughout the volume, as well as in a thin layer, near the free surface of the medium as well as at different depths in such a way that the free surface of some POS entered the free surface formed by the continuous phase of the medium. Furthermore, it was assumed that the pseudo surface phase consisted of the free surface of the medium, the outer osmotic cell wall (OOPM), and the inner thin elastic cytoplasmic membrane (IECM) of the bacterial cell [58]. The PSP geometric shape and physical parameters depend on the shape and physical properties of the surrounding phases, respectively. The sigma phase (Σ—phase) exists in multiphase systems and participates in mass and heat transfer between phases [59]. The free surface in a real three-phase system is characterized by a complex geometry, and in the theoretical model, it can be treated as a regularly undulating free surface. RMF, medium free surface area, and OS mass flow are the main quantities affecting microorganisms on the medium surface. The physical nature of the free surface of the medium has the greatest influence on the growth of bacterial colonies. Thus, an increase or decrease in bacterial colony counts depends on the free surface with OS with activity controlled by available external factors. An example presenting one of such interactions is manifested by the fact that RMF significantly affects the physical state of the interfacial surface between the fluid and the solid phase in the technical process of manufacturing crystals from a liquid metal alloy [60].

In the first stage, we analyzed the free surface activity of the medium exposed to RMF. This type of analysis is of significant practical importance in microbiological processes, which are initially activated on such surfaces no matter whether the medium is controllably prepared or constitutes a free surface of any physical state. It should be kept in mind that in technical processes using RMF, magnetic fluids (medium) with a defined set of physical parameters of the electromagnetic field were used [36,37]. MF interacts with a multicomponent or dispersed system when volumes of these systems contain free electric charges, magnetic particles with their associated magnetizing effect, and perform an ability to accumulate and dissipate radiant magnetic energy [61,62,63]. We assumed that the magnetic susceptibility of OS can be generally defined, similar to any physical substance, by essential electromagnetic parameters, such as electrical conductivity and magnetic susceptibility [64]. In order to formulate the theoretical model which includes interaction between a medium and a bacterial colony, both exposed to RMF, we made specific assumptions concerning the geometric configuration as well as the mechanical and electromagnetic properties of the free surface of the medium. It was assumed that RMF influences OS if the investigated medium is adequately similar to magnetic fluid with defined magnetic properties. Furthermore, based on our assumptions, the experimental OS in our study contained chemical elements sensitive to MF, and hence, small, isolated POS with constant magnetic permeability should be treated as isolated magnetic particles of organic substance (MPOS). Moreover, we also assumed, that that the MPOS on a surface with a thin layer of the medium is influenced by RMF.

In the theoretical model, we assumed that the free surface of the medium has cylindrical-shaped craters filled with MPOS, through which the outflow of OS is supplemented by a diffusive inflow of this substance from the interior of the medium. These craters are continually refilled with OS at a rate that depends on the depth of POS hiding under the free surface of the medium and on the rate of the mass diffusion process. This phenomenon repeats itself until the organic substance is exhausted or the bacterial colony is destroyed. In a microvolume of randomly dispersed craters filled with MPOS with their own certain electromagnetic parameters, MF induction lines penetrate OS, i.e., diffuse through the substance into the surface layer to a depth determined by the MF diffusion coefficient or magnetic kinematic viscosity coefficient depending on the electrical conductivity of OS, magnetic susceptibility of OS and RMF frequency [61,65,66]. Finally, MPOS of an equal number of poles (magnetic charges) of opposite polarity appears in the craters [67].

RMF orientates MPOS and forms magnetic dipoles. A collection of dipoles sends out a packet of induction lines and the volume of fluid in the crater becomes magnetized. These dipoles generate their inductive heterogeneous spin of MF inside OS whose magnetic force can move MPOS in a spin motion opposite to the direction of the external induction vector of RMF. MFs and especially RMFs, force a cylindrical volume of molten material into a uniform rotational motion with an azimuthal driving force proportional to the magnitude of RMF induction and frequency, electrical conductivity, and the diameter of the cylindrical volume of molten fluid [68]. However, the sample spins more slowly than the frequency of MF due to the viscosity of the surrounding fluid and the inertia of the cylindrical volume [65,69]. Hence, the magnetic dipoles of MPOS in a vortex motion can carry from the free surface of the medium an attached thin layer of mass of the medium inertial phase, an antimicrobial agent as well as other components present in the medium. Nevertheless, it can also be assumed that in the crater, a complex system of forces generated by various physical phenomena (collision, friction, oscillation, inertia) acts on their MPOS spin motion, and especially in the presence of small volume craters, the phenomena of non-magnetic nature can be neglected.

In the initial phase of the metabolic process, bacterial enzymes and RMF act on the local free surface area of small craters with POS organic substance. Surface tension in this area is then lowered and this area becomes an accessible source of OS. In view of the difference in organic substance concentration between the open free surface area of the medium as well as the bacterial cell, there is an outflow of an organic substance into the OOPM and thus through the IECM into the cell. As a result, the vital activity of bacteria increases, and the interaction of external RMF and induced internal RMF with magnetic dipoles of MPOS hidden in the layer under the free surface of the medium can decrease the surface tension of the medium-free surface partially interrupted by active craters. In turn, such a phenomenon contributes to an increase in the number of open OS-mass craters on this surface and an increase in OS influx into bacterial cells. Therefore, as a result of the RMF activity, MPOS moving through PSP reach OOPM of the bacterial cell, where, after penetrating IECM, they are absorbed by the cell. At a given point in the process, the metabolic activity of the bacteria increases, the surface organic substance content decreases, and the diffusive influx of OS from deeper layers of the medium to the layer below the free surface increases. MPOS can attach to TA molecules introduced into the medium. Therefore, the simultaneous presence of bacterial toxins induces counter-current movement of these chemicals. MPOS and TA molecules leave the crater and diffusely penetrate PSP to enter the pore target wall and then the bacterial cell. In the opposite direction, using the same type of movement, some of the toxins leave the bacterial cell and penetrate into some of the craters on the free surface contaminating the medium. Some of the toxins may remain in the cell, affecting the vital function of bacteria. These phenomena are dynamic processes. The biological resistance of the medium surface can be weakened by an introduction of a selective chemical substance into the layer under the free surface of the medium that lowers the surface tension. Ideally, this substance should also exhibit antimicrobial properties to some extent.

The introduction of chemically crafted small magnetic particles into the medium can modify the surface layer [70,71]. Such modification can be made by introducing inorganic Fe nanoparticles, Fe_3_O_2_, Fe_3_O_4_ [72,73,74], or magnetic surface-modified particles dispersed in organic polymers to form a magnetic composite [75]. Bacteria can also be magnetically modified to form magnetotactic bacteria by the addition of immunomagnetic nano- and microparticles to the suspension with bacterial cells [46,49,50,51,52,53]. Microorganisms can be biomineralized via the adsorption of magnetic fluids (aqueous electrolytes with ions Ni^2+^, Cu^2+^, Cd^2+^, or Ag^2+^ as adsorbents, or can synthesize intracellular ferromagnetic crystalline with nanocrystals of magnetite (Fe_3_O_4_) or greigite (Fe_3_S_4_) [76,77,78,79,80,81,82]. Accumulation of a large number of nanocrystals of ferromagnetic metals in OOPM of the cell modifies its surface. In this case, one would expect a change in the OOPM of the bacterial cell, and the result would be either a change in mass and heat transport or in the elasticity of the bacterial cell membrane, which, like any physical membrane, is sensitive to external force actions of RMF. In this context, magnetotactic bacteria in RMF behave similarly to a magnetic dipole in suspension [50,51]. RMF and MPOS mass are sources that activate biological microparticles enclosed by the inner elastic membrane of the bacterial cell, where they are randomly distributed. The magnetic induction vector flux energetically activates MPOS present in OOPM pores and penetrates the interior of the bacterial cell.

Under the influence of MF, negatively charged biological microparticles and their own electromagnetic parameters randomly dispersed in the bacterial cell, form a material rotating “chain” oriented in the direction of MF lines. A similar phenomenon of chain formation occurs for magnetic particles Fe, Fe_3_O_2_, Fe_3_O_4_ under the influence of RMF [29,81]. An electrically conductive “pathway” is formed and consists of a chain of biological microparticles, IECM and OOPM, as well as craters on the free surface of a MPOS-filled medium. An interaction between RMF and an electrically conductive “pathway” generates a microcurrent in the MPOS flow and the action of RMF on the microcurrent results in an elementary volume micro electromagnetic force that depends on the physical electromagnetic parameters of OS, frequency of the external RMF mechanically acting on the crater, OOPM, and IECM of the bacterial cell. It compresses the bacterial cell and elongates it in the direction of the MF induction vector, and with a high modulus of this force, the chain of molecules, as well as the bacterial cell, can get ruptured. Hence, like any elastic membrane, bacterial cell membranes can retain their original shape or get deformed under normal surface force. The mechanical properties of bacterial cells are described by Young’s modulus and bending rigidity. If the stiffness of a bacterial membrane can be determined by the tensile elasticity and the stress/strain curve in the linear region, the bacterial cells will deform, whereas the removal of the load causes the membrane to return to its pre-load state [82]. At low-frequency RMF, an elementary volumetric electromagnetic microgrid is generated. It deforms the inner thin elastic membrane and generates slight oscillatory chain movement of particles in the cell, narrowing the pore on OOPM and the width of channels on the free surface of the medium, which in turn affects the resultant rate of the MPOS movement from the medium into the bacterial cell. When RMF frequency increases, the stress–strain curve is no longer linear. This results in micro-permanent dynamic deformation of the inner thin elastic membrane of the bacterial cell [83]. The highly increased intensity of RMF and simultaneous low elasticity of the inner thin elastic membrane contribute to the deformation which may irreversibly destroy the cell. Therefore, it can be expected that a sudden jump in high field stress will result in a mechanical rupture of the bacterial cells. 

## 4. Materials and Methods

### 4.1. Reference Strain, Culture Condition, and Preparation of Staphylococcal Suspension

Prior to the experiment, the *S. aureus* FRI913 strain (harboring *sea*, *sec*, *sel* genes) [84] was cultivated for 18 h at 37 °C on Columbia agar with 5% sheep blood (bioMérieux, Warsaw, Poland). Then, a single colony was transferred into fresh Mueller–Hinton broth (Merck Life Science, Poznan, Poland) and incubated at 37 °C overnight. After incubation, the bacterial culture was centrifuged (5000× *g* for 10 min), washed three times using phosphate-buffered saline (PBS, pH 7.4), and adjusted to 1.5 × 10^8^ CFU/mL density. The obtained suspension was used in all further experiments.

### 4.2. Minimum Inhibitory Concentration (MIC) Assay

The MIC of TA (99.0% purity; Merck Life Science, Poznan, Poland) was determined using the agar dilution method in compliance with the Clinical and Laboratory Standards Institute guidelines [85] with a few modifications, as described in our previous study [86]. Briefly, Mueller–Hinton agar (MHA; Merck Life Science, Poznan, Poland) was mixed with Tween 80 (1%, *v*/*v*; Merck Life Science, Poznan, Poland) and selected TA concentrations (0.31–10%, *v*/*v*). Next, the staphylococcal suspension was transferred onto prepared media (10^4^ CFU per spot; 32 spots per plate) and incubated for 18 h at 37 °C.

MIC_100_ of TA was evaluated after incubation and recognized as the lowest concentration of TA which completely inhibited the visible growth of *S. aureus* FRI913 on the MHA. The MIC_50_ value (which is required to inhibit the growth of 50% staphylococcal cells) was calculated proportionally to the MIC_100_ value. MHA supplemented with 1% (*v*/*v*) Tween 80 was used as a positive growth control for *S. aureus* FRI913. Concurrently, a sterility control was performed. The experiment was conducted in duplicates. The results were expressed in mg/mL.

The following culture media were prepared: MHA (medium A), MHA supplemented with 1% (*v*/*v*) Tween 80 (medium B), and MHA supplemented with 1% (*v*/*v*) Tween 80, and MIC_50_ of TA (medium C). The media were used in all further stages of the experiment.

### 4.3. Rotating Magnetic Field Generator

**Figure 4:**
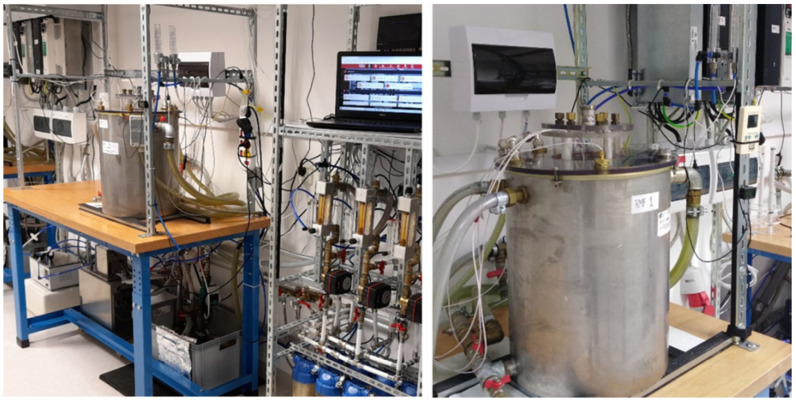
RMF generator with monitoring and control equipment.

The design of the RMF generator was based on a three-phase, four-pole stator with an internal diameter of 16 cm and a height of 20 cm, consisting of twelve groups of three coil sets (Figure 4). The frequency of the alternating current (AC) provided to the RMF generator was regulated using a Unidrive M200 inverter (Control Techniques, Nidec Industrial Automation, Poznan, Poland). The temperature in the RMF reactor chamber was controlled using a water thermostat (KISS K6, Huber, Germany) equipped with a set of temperature probes (accuracy range ± 1.0 °C). The distribution of magnetic induction (*B*) in the reactor chamber was performed at an initial voltage of AC of 100 V and AC frequencies of 5 Hz and 50 Hz using the Ansys Maxwell simulation software ver. 19.1 (Ansys, Inc., Canonsburg, PA, USA) and using a teslameter (SMS-102, Asonik, Tuczno, Poland). 

### 4.4. Exposure of S. aureus FRI913 to RMF

**Figure 5:**
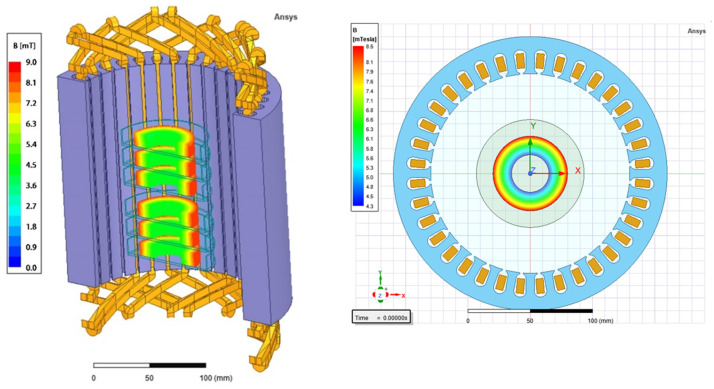
Arrangement and location of the Petri dishes in RMF reactor chamber. The place from which the bacteria were harvested for further tests and analyzes has been marked as a colored circle; different colors show the distribution of the magnetic induction depending on its value.

In the first stage of the experiment, the staphylococcal suspension (100 μL) was introduced on A–C media, spread using a sterile spreader, and incubated for 12 h at 37 °C in the water bath (unexposed to RMFs) or in RMF generators at two different RMF frequencies: RMF I: 5 Hz, RMF II: 50 Hz. The arrangement and location of the Petri dishes in the RMF reactor chamber are graphically presented in Figure 5.

In the second stage of the experiment (after 12 h of incubation), media A–C incubated in a water bath as well as exposed to RMFs were transferred to the incubator, and the cultures were incubated for the next 12 h (37 °C). In the RMF generator, a water bath as well as incubator, the same temperature (37 °C) and relative humidity RH (60%) were maintained throughout the entire experiment. Bacteria cultured on medium A and unexposed to RMFs were used as the control. A schematic diagram of the experimental setup is graphically presented in Supplementary Materials, Figure S1.

### 4.5. RNA Isolation

After 24 h of incubation on A–C media unexposed and exposed to RMFs, bacteria were harvested, adjusted to 1.2 × 10^9^ CFU/mL, transferred to 1.5 mL tubes, and washed three times using PBS. To ensure uniform exposure to the RMF, the bacteria were harvested from the zone 1.5 cm from the edges of the plate and 3 cm from the center, marked with a circle in Figure 5. The supernatant after the first washing was preserved for the ELISA test. Then, 400 µL of acid guanidinium thiocyanate-phenol-chloroform-based reagent (A&A Biotechnology, Gdansk, Poland) was added to each bacterial pellet and frozen at −20 °C to prevent RNA degradation. The RNA isolation procedure was carried out on the next day. After thawing, 50 mg of glass beads (diameter 150–212 μm; Merck, St. Louis, MO, USA) was added to the samples. Three cycles of beating (2 min each), with 1 min incubation on ice within cycles, were carried out in the Tissue Lyser LT (Qiagen, Hilden, Germany) at 4 °C. Total RNA was isolated with the RNA Isolation Mini Kit (A&A Biotechnology, Gdansk, Poland) in accordance with the manufacturer’s instruction. Next, RNA was dissolved in 50 µL of RNase-free water (A&A Biotechnology, Gdansk, Poland). The RNA concentration was measured using a spectrophotometer (DeNovix DS-11 FX, Wilmington, DE, USA), and the purity of the isolated RNA was assessed spectrophotometrically (A_260_ nm/A_280_ nm). Contaminated DNA was removed using RNase-free DNase I (Merck, St. Louis, MO, USA) digestion for 1 h at 37 °C. The integrity of RNA was evaluated by electrophoresis on 1% (*w/v*) agarose gel. RT-PCR was performed using SuperScript™ III Reverse Transcriptase (Invitrogen, Carlsbad, CA, USA). For cDNA synthesis, 100 ng of RNA was mixed with 1 µL of 50 µM Random Hexamers and 1 µL 10 mM dNTP Mix, heated to 65 °C for 5 min, and incubated on ice for 1 min. In the next step, 4 µL of 5XFirst-Strand Buffer, 1 µL of 0.1 dithiothreitols, 1 µL of RNaseOUT™ Recombinant RNase Inhibitor, and 1 µL of SuperScript™ III RT (Invitrogen, Carlsbad, CA, USA) were added and incubated at 50 °C for 60 min. The reaction was inactivated by heating at 70 °C for 15 min.

### 4.6. Quantitative Real-Time PCR (qPCR)

qPCR was carried out on a CFX Connect™ Real-Time System (Bio-Rad, Herkules, CA, USA), using SsoFast EvaGreen Supermix (Bio-Rad, Herkules, CA, USA). The reaction mixture contained 1 µL of cDNA template, 0.5 μM of each primer (listed in Supplementary Materials, Table S4), 10 µL of SsoFast EvaGreen Supermix, and water up to 20 µL. Reaction mixtures were initially incubated for 30 s at 95 °C, followed by 35 cycles at 95 °C for 10 s, and 15 s at 62 °C. The specificity of PCR was evaluated by a melt curve analysis at a temperature ranging from 95 to 58 °C performed for each reaction. Residual DNA contamination was checked in each RNA sample by running no-RT controls. All samples were analyzed in triplicate.

The housekeeping gene was selected from 8 candidate genes, including *rpoB*, *16S rRNA*, *gyrB*, *recA*, *rho*, *pta*, *rplD*, and *tpo* based on its expression stability [87]. The BestKeeper software (https://www.gene-quantification.de/bestkeeper.html, accessed on 5 January 2022) was used to assess the variability of housekeeping genes. As a positive control of the reaction, *S. aureus* FRI913 DNA was used. PCR efficiencies determined for all tested primer pairs were above 96%. Relative transcript levels were calculated according to results obtained by Pfaffl [88]. Data analysis was carried out using the Bio-Rad CFX Manager (Bio-Rad, Herkules, CA, USA) software.

### 4.7. Sandwich ELISA

#### 4.7.1. Samples Preparation

After 24 h of incubation on A-C media unexposed and exposed to RMFs, 1 mg of the bacterial biomass was collected from each plate (to ensure uniform exposure to the RMF, the bacteria were harvested from the zone 1.5 cm from the edges of the plate and 3 cm from the center, marked with a circle in Figure 5) and mechanically lysed by homogenization with 0.5 mg of glass beads in 1 mL of supernatant after first washing the pellets, in the Tissue Lyser LT (Qiagen, Hilden, Germany). After centrifugation (2000× *g*, 5 min at 4 °C) supernatants were preincubated with 20% normal rabbit serum overnight at 4 °C (in order to bind protein A) and diluted 4 times in PBS supplemented with 0.1% (*v*/*v*) Tween 20 (Merck, St. Louis, MO, USA).

#### 4.7.2. Sandwich ELISA Preparation

Sandwich ELISA was performed as described by Lis et al. [89]. Rabbit polyclonal anti-SEA and anti-SEC antibodies were purchased from Acris Antibodies (Herford, Germany). Rabbit polyclonal IgG antibodies against SEL were prepared by immunizing 3-month-old white male New Zealand rabbits. Recombinant proteins for immunization were obtained with a technique previously described by Podkowik et al. [84]. Briefly, the *sel* gene was cloned into the pET-22b plasmid vector and expressed in *E. coli* rosetta competent cells (Merck, St. Louis, MO, USA).

Expression of rSEL was performed using isopropyl β-D-1-thiogalactopyranoside (IPTG; Merck, St. Louis, MO, USA), then induction and purification was conducted on His-Select Cobalt Affinity Gel (Merck, St. Louis, MO, USA), with on-column refolding. The anti-rSEL antibody in the serum was purified using an affinity column. The second layer of antibodies was prepared by antibody biotinylation with biotin N-hydroxysuccinimide ester (Merck, St. Louis, MO, USA), instead of coupling the antibody with horseradish peroxidase. The ELISA was carried out in 96-well microtiter plates. Each well was coated with 50 µL of antibody (2 µg/mL) specific for the tested SEs in 0.05 M carbonate–bicarbonate buffer (pH 9.6; Merck, St. Louis, MO, USA) at room temperature overnight. A standard curve was prepared by using recombinant proteins: SEC (rSEC), SEL (rSEL) [84], and SEA (rSEA), purchased from Merck (St. Louis, MO, USA). Antibody-coated wells were blocked with 250 µL/well of PBS/0.1% bovine serum albumin for 1 h at 25 °C. Next, 100 µL/well of samples or standards were added and incubated at 25 °C for 2 h. After washing, biotinylated antibodies were added to each well and incubated at 37 °C for 1 h. Biotinylated antibody was detected with the conjugate of HRP-streptavidin (Merck, St. Louis, MO, USA). 3,3′,5,5′-tetramethylbenzidine (TMB, Merck, St. Louis, MO, USA) was used as a substrate for HRP. The absorbance at 405 nm was read with a microplate reader (Spark Tecan, Männedorf, Switzerland).

In order to determine SE concentrations, a 4-parameter logistic curve was prepared using a known concentration of rSEC, rSEL, and SEA. Data were analyzed using GraphPad Prism software (GraphPad Software Inc., La Jolla, CA, USA).

### 4.8. Statistics

The statistical significance of the results was determined using a one-way ANOVA test. Spearman’s rank correlation coefficient was used to detect relations between SE mRNA expression and SE protein production. The following values: * *p* < 0.05, ** *p* < 0.01, *** *p* < 0.001 were considered statistically significant. Statistical analyses were performed using Statistica version 12 (StatSoft Inc., Cracow, Poland) and GraphPad Prism 5.02 (GraphPad Software Inc., San Diego, CA, USA).

## 5. Conclusions

The obtained results allowed us to conclude that there were no significant differences in the expression of regulatory and SE genes in bacteria simultaneously cultured on a medium supplemented with TA and exposed to RMF at the same time in comparison to the control (unexposed to TA and RMF). In contrast, when the bacteria were cultured on a medium supplemented with TA but were not exposed to RMF or when they were exposed to RMF of 50 Hz (but not to TA), a significant increase in *agrA* and *sea* transcripts as compared to the unexposed control was found. Moreover, the decreased level of *sec* transcripts in bacteria cultured without TA but exposed to RMF of 50 Hz was also revealed. In turn, a significant increase in SEA and decrease in SEC and SEL production was observed in bacteria cultured on a medium supplemented with TA and simultaneously exposed to RMFs. It can be concluded, that depending on SE and regulatory genes’ expression, as well as production of SEs, the effect exerted by the RMF and TA may be positive (i.e., manifests as the increase in SE and/or regulatory gene expression of SEs production) or negative (i.e., manifests as the reduction in both aforementioned features) or none. The experimental results suggest that the above type of variables could be usefully, e.g., in the control of *S. aureus* gene expression, which in turn can have an enormous impact in the food industry (e.g., food safety and quality). However, the use of specific parameters and factors to determine the optimal conditions of such a process still require further development.

## Data Availability

Not applicable.

## Supplementary Materials

The following supporting information can be downloaded at: https://www.mdpi.com/article/10.3390/ijms23116327/s1. References [90,91] are cited in the Supplementary Materials.
