# Supplementary material for: Regulatory and Enterotoxin Gene Expression and Enterotoxins Production in Staphylococcus aureus FRI913 Cultures Exposed to a Rotating Magnetic Field and trans-Anethole
Source: Int J Mol Sci. 2022 Jun 6;23(11):6327. doi: 10.3390/ijms23116327 (PMC9181688; doi:10.3390/ijms23116327)
Supplement: Supplementary file 1 [file ijms-23-06327-s001.zip › ijms-1737784-supplementary.pdf]

Table S1. Statistical analysis of eight candidate housekeeping genes.

Statistics	Gene name							
	16S rRNA	*pta*	*rho*	*gyrB*	*recA*	*rplD*	*rpoB*	*tpo*
GM [Cq]	22.77	26.58	30.22	26.33	31.43	30.95	29.96	27.19
Min [Cq]	21.81	25.03	25.48	24.58	26.30	27.18	24.76	24.90
Max [Cq]	23.60	28.00	33.76	27.41	36.70	34.09	34.56	30.18
SD [± Cq]	0.44	0.90	2.96	1.04	3.17	2.05	2.66	1.92
CV [% Cq]	1.95	3.38	9.73	3.95	10.01	6.59	8.83	7.03
CC [r]	0.930	0.877	0.811	-0.034	0.595	-0.068	-0.133	0.669
p value	0.001	0.001	0.001	0.915	0.041	0.830	0.683	0.017

Cq – cycle of quantification; GM – geometric mean; Min – minimal value; Max – maximal value; SD – standard deviation; CV – coefficient of variance; CC [r] – correlation coefficient between each candidate and the BestKeeper index. BestKeeper performs repeated pairwise correlation and regression analyses for a given gene with all other genes.

Table S2. Cycle of quantification (Cq) obtained in this study.

Samples	Media	Cq ± SEM						
		16S rRNA	*sea*	*sec*	*sel*	*agrA*	*hld*	*rot*
Unexposed to the RMFs (control)	A	18.15±0.24	29.17±0.17	27.38±0.30	22.83±0.31	22.75±0.23	23.75±0.29	25.89±0.13
	B	19.59±0.13	30.85±0.25	29.01±0.59	24.09±0.65	23.90±0.53	25.02±1.0	26.95±0.18
	C	18.23±0.39	28.44±0.44	27.80±0.35	22.67±0.15	21.67±0.24	23.01±0.37	25.62±0.65
Exposed to the RMF I (5 Hz)	A	19.25±0.08	30.22±0.69	29.22±0.39	24.70±0.26	23.76±0.66	24.42±0.18	27.38±0.36
	B	19.67±0.22	30.17±0.13	28.91±0.51	20.61±0.84	23.68±0.21	25.10±0.23	26.63±0.57
	C	19.41±1.04	31.27±0.19	29.27±0.19	23.14±0.92	23.74±0.18	24.90±0.11	26.90±0.11
Exposed to the RMF II (50 Hz)	A	16.97±0.03	24.90±0.68	28.31±0.36	21.82±0.22	20.42±0.38	21.85±0.06	23.90±0.56
	B	18.45±0.49	26.68±0.10	27.80±0.97	23.51±0.24	21.70±0.15	23.81±0.26	25.28±0.23
	C	19.61±0.39	30.68±0.41	30.15±0.65	24.80±0.56	23.73±0.35	24.15±0.35	27.08±0.16

SEM – standard error of measurement; RMF – rotating magnetic field; A – Mueller-Hinton agar (MHA) non-supplemented with *trans*-anethole; B – MHA supplemented with 1% (*v/v*) Tween 80; C – MHA supplemented with 1% (*v/v*) Tween 80 and subinhibitory concentration (MIC_{50}) of *trans*-anethole.

Table S3. Correlation analysis between staphylococcal enterotoxin (SE) genes expression and SEs production.

Samples	SE	Media	Spearman coefficent [r]	*p* value
Unexposed to the RMFs (control)	*sea vs.* SEA	A+B+C	0.7622	0.0040
Exposed to the RMF I (5 Hz)		A+B+C	0.7566	0.0044
Exposed to the RMF II (50 Hz)		A+B+C	0.5359	0.0725
Unexposed to the RMFs (control)	*sec vs.* SEC	A+B+C	0.8392	0.0006
Exposed to the RMF I (5 Hz)		A+B+C	-0.2487	0.4357
Exposed to the RMF II (50 Hz)		A+B+C	0.0541	0.8674
Unexposed to the RMFs (control)	*sel vs.* SEL	A+B+C	0.5315	0.0754
Exposed to the RMF I (5 Hz)		A+B+C	-0.4685	0.1245
Exposed to the RMF II (50 Hz)		A+B+C	-0.3986	0.1993

RMF – rotating magnetic field; A – Mueller-Hinton agar (MHA) non-supplemented with *trans*-anethole; B – MHA supplemented with 1% (*v/v*) Tween 80; C – MHA supplemented with 1% (*v/v*) Tween 80 and MIC_{50} of *trans*-anethole.

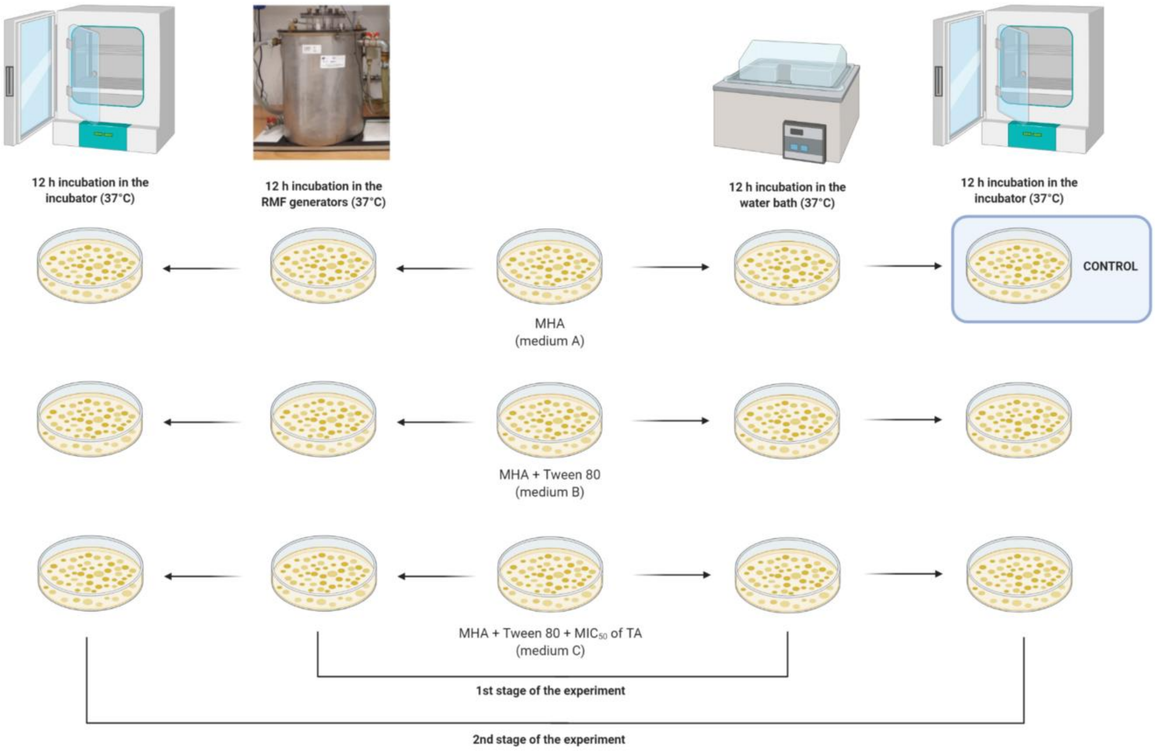

Figure S1. Schematic diagram of the experiment. Created with BioRender.com.

Table S4. Primer sequences for real-time quantitative PCR analysis.

Gene	Sequence	Amplicon length (bp)	References
sea	F: 5′-CAG CAT ACT ATA TTG TTT AAA GGC-3′<br>R: 5′-CCT CTG AAC CTT CCC ATC-3′	400	[1]
sec	F: 5′-CTC AAG AAC TAG ACA TAA AAG CTA GG-3′<br>R: 5′-TCA AAA TCG GAT TAA CAT TAT CC-3′	271	[1]
sel	F: 5′-TAA CGG CGA TGT AGG TCC AGG-3′<br>R: 5′-CAT CTA TTT CTT GTG CGG TAA C-3′	383	[1]
agrA	F: 5′-CCT CGC AAC TGA TAA TCC TTA TG-3′<br>R: 5′-ACG AAT TTC ACT GCC TAA TTT GA-3′	127	[2]
hld	F: 5′-TAA GGA AGG AGT GAT TTC AAT GG-3′<br>R: 5′-GTG AAT TTG TTC ACT GTG TCG AT-3′	90	[2]
rot	F: 5′-TGC AGT ATT TCA ACC ACA CAC-3′<br>R: 5′-GTA TCG TTA ATG CGC CAG T-3′	140	[2]

References

1. Park, J.Y.; Fox, L.K.; Seo, K.S.; McGuire, M.A.; Park, Y.H.; Rurangirwa, F.R.; Sischo, W.M.; Bohach, G.A. Detection of classical and newly described staphylococcal superantigen genes in coagulase-negative staphylococci isolated from bovine intramammary infections. *Vet. Microbiol.* **2011**, *147*, 149–154, doi:10.1016/j.vetmic.2010.06.021.
2. Schubert, J.; Podkowik, M.; Bystroń, J.; Bania, J. Production of staphylococcal enterotoxins D and R in milk and meat juice by *Staphylococcus aureus* strains. *Foodborne Pathog. Dis.* **2017**, *14*, 223–230, doi:10.1089/fpd.2016.2210.
